# Supplementary material for: Proteome-Wide Analysis Reveals TFEB Targets for Establishment of a Prognostic Signature to Predict Clinical Outcomes of Colorectal Cancer
Source: Cancers (Basel). 2023 Jan 25;15(3):744. doi: 10.3390/cancers15030744 (PMC9913156; doi:10.3390/cancers15030744)
Supplement: Supplementary file 1 [file cancers-15-00744-s001.zip › cancers-2083394-supplementary.pdf]

Supplementary Materials: Proteome-Wide Analysis Reveals TFEB Targets for Establishment of a Prognostic Signature to Predict Clinical Outcomes of Colorectal Cancer

Zi-Jia Huang, Sheng Zhu, Zi-Qin Han, Chen Li, Jun-Ze Liang, Yang Wang, Shuixing Zhang and Jing Zhang

Supplementary materials and methods for qRT-PCR analysis

Total RNA was extracted using the HiPure Total RNA Mini Kit (Magen Biotechnology, Guangzhou, china) according to the manufacturer's protocol. cDNA was generated using the Hifai Ⅱ 1st Strand cDNA Synthesis SuperMix (Yeasen Biotechnology, Shanghai, China). qPCR was performed using a SYBR green mixture (Yeasen Biotechnology) and Applied Biosystems StepOne Real-Time PCR System (Thermo Fisher Scientific , Waltham, MA, USA). Primers: SMPD1 forward, 5′-GCTGGCTCTATGAAGCGATGGC-3′and reverse, 5′-AGAGCCAGAAGTTCTCACGGGA-3′. NDRG1 forward, 5′-AGCTCGTCAGTTCACCATCC-3′and reverse, 5′-GAGTACGCGGGGCTACAAAG-3′. LAMP2 forward, 5′-GGCAATGATACTTGTCTGCTGGC-3′and reverse, 5′-GTAGAGCAGTGTGAGAACGGCA-3′. Actin forward, 5′-ACGTGGACATCCGCAAAG-3′and reverse,5′-GACTCGTCATACTCCTGCTTG-3′.

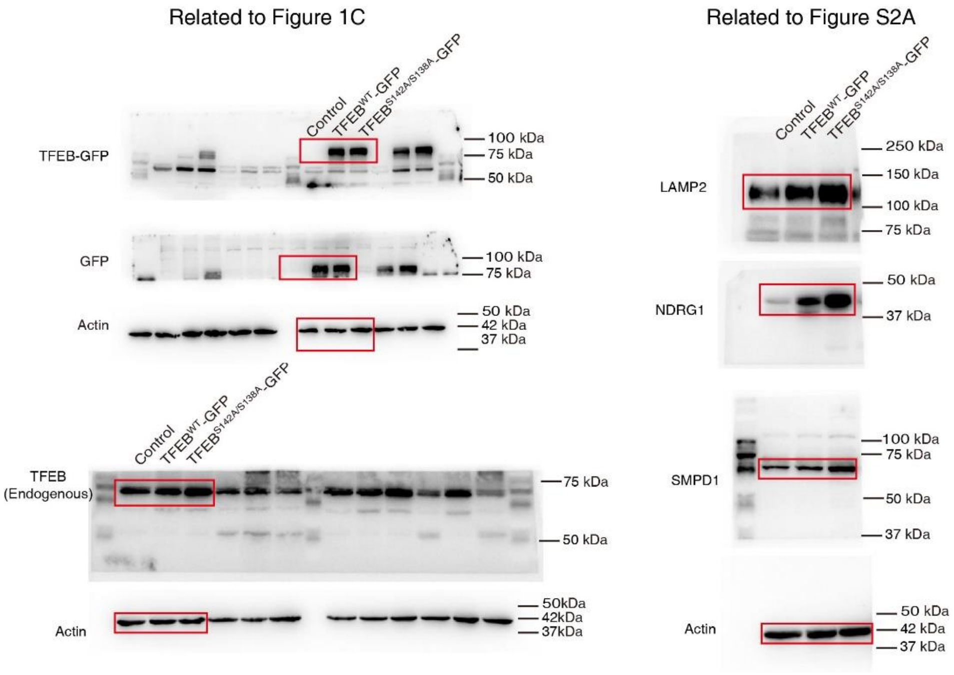

Figure S1. Uncropped Western Blots for Figure 1 and FigureS2A.

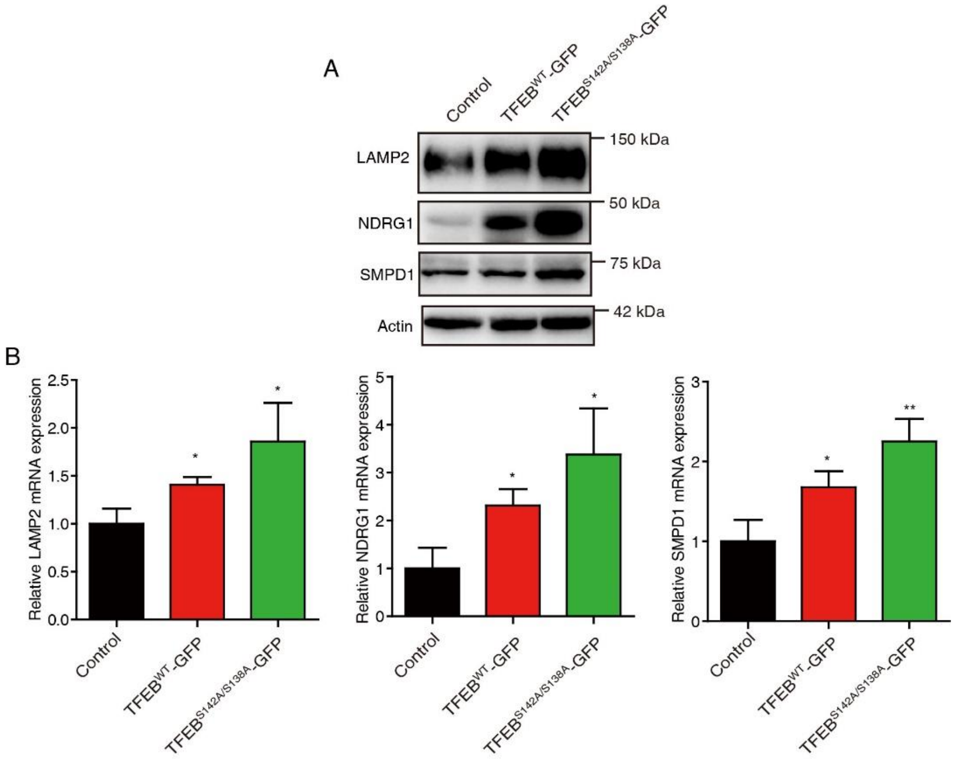

Figure S2. Verification of randomly selected DEPs. (A) Western Blots of LAMP2, NDRG1 andS-MPD1 in the indicated DLD1 cells. Actin was used as loading control. Uncropped immunoblots are provided in the Figure S1. (B) qRT-PCR analysis of mRNA levels of LAMP2, NDRG1 andS-MPD1.

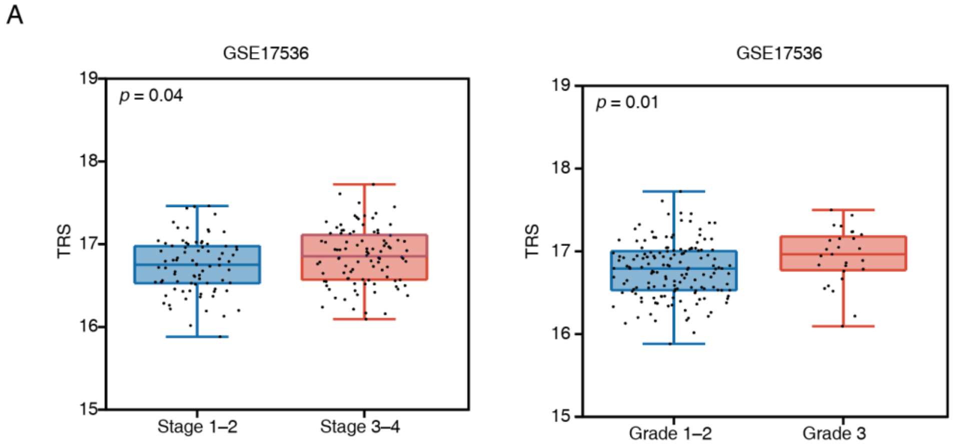

Figure S3. Distribution of TRS in stage and grade.

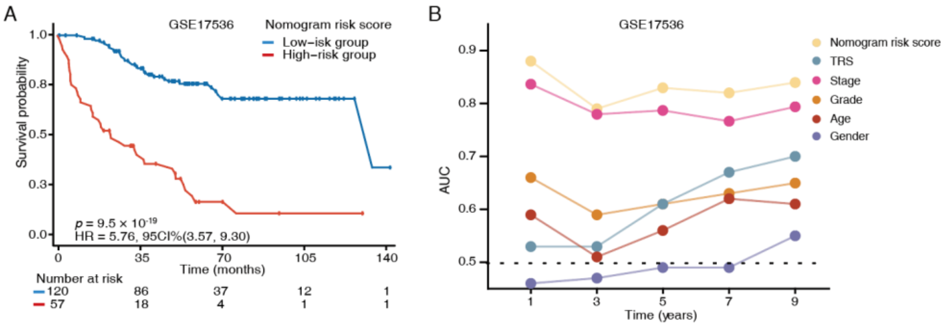

Figure S4 The external validation of the nomogram (A). Kaplan–Meier curves of OS between high-risk and low-risk patients in the GSE17538 cohort (B) tROC analysis assessed the accuracy of nomogram.

Table S1. The quantifiable protein list in $TFEB^{WT}$, $TFEB^{S142A/S138A}$ and control datasets.

Table S2. The differential expressed proteins in $TFE^{BS142A/S138A}$ vs $TFEB^{WT}$ and $TFEB^{WT}$ vs control datasets.

Table S3. The protein list of TFEB targets (Fold change: $TFEB^{S142A/S138A}$ vs $TFEB^{WT}$ > $TFEB^{WT}$ vs control > 1.2; $p < 0.05$).

Table S1–S3. are provided separately, attached as Excel files
